# A neonate born to an infected COVID‐19 mother was tested positive just 24 hours after its birth

**DOI:** 10.1002/ccr3.3913

**Published:** 2021-02-10

**Authors:** Roya Arif Huseynova, Latifa A. Bin Mahmoud, Oqtay Huseynov, Mishary Almalkey, Ameen Amer Almotiri, Haider Hussein Sumaily, Adli AbdelRahim

**Affiliations:** ^1^ Neonatology Department King Saud Medical City Riyadh Saudi Arabia; ^2^ Azerbaijan Medical University Baku Azerbaijan; ^3^ Pediatric Department King Saud Medical City Riyadh Saudi Arabia

**Keywords:** coronavirus, COVID‐19, infection, newborn, SARS‐CoV‐2

## Abstract

Given the importance of possible vertical transmission, it is essential to study all neonates delivered from COVID‐19 positive mothers to analyze the route of transmission of infection that will optimize the universal testing for SARS‐CoV‐2 and minimize the risk of disease in neonates.

## 1 INTRODUCTION

Coronavirus disease (COVID‐19) is a highly contagious disease with a rapid increase in cases and mortality all over the world. We report a neonate born to the pregnant woman with COVID tested positive for SARS‐CoV‐2 infection just 24 hours after its birth.

We report a 37 weeks’ gestation newborn confirmed by real‐time reverse‐transcription polymerase chain reaction (RT‐PCR) to be positive after a nasopharyngeal swab was taken at 24 hours of age.

Coronavirus disease (COVID‐19) is a global health hazard caused by severe acute respiratory syndrome coronavirus 2 (SARS‐CoV‐2). COVID‐19 was first detected in Wuhan's city, the capital of Hubei Province, China, in December 2019.^1^ One of the primary risk factors of COVID‐19 infection is considered close contact with an infected person within 14 days of symptoms onset.

Several articles report the outcomes of pregnant women infected in the present course of the COVID‐19 pandemic; these include the risk of miscarriages, preterm delivery, and perinatal mortality.^2^, ^3^

Evidence suggesting transmission for SARS‐CoV‐2 from mother to her baby was reported in the previous studies; however, is it an intrauterine or intrapartum or environmental way of exposure is still not clear.

It is essential to study all neonates delivered from COVID‐19 positive mothers to analyze the route of transmission of infection that will optimize the universal testing for SARS‐CoV‐2 and minimize the risk of disease in neonates.

## 2 CASE REPORT

The index case mother is a 16‐year‐old primigravida pregnant woman who presented to the maternal emergency department on June 13th, 2020, at 17:0 due to premature rupture of membrane (PROM) for 8 hours. Her previous medical history was unremarkable of any medical or pregnancy‐related illnesses. She was following up on her pregnancy in a private hospital. There was no history of suspected or confirmed individuals with COVID‐19 among her family members.

The mother was admitted to the prenatal ward for observation and started on intravenous Ceftriaxone because of PROM.

Her initial assessment was body temperature 36.5°C, blood pressure 112/66 mm Hg, respiratory rate 20 breaths per minute, heart rate 118 beats per minute, and oxygen saturation 95% in room air. She did not have any upper respiratory symptoms (cough, sneezing, or sputum). Fetal heart monitoring was reassuring between 130 and 140 beats per minute, with no signs of abnormality.

Blood tests showed white blood cells count 10.8 x109 cells /L, neutrophil 7.67x109 cells/L, hemoglobin level 10 g/dl, and platelet count 194 x109 cells /L; coagulation profile (prothrombin time 11.8 s, partial thromboplastin time 28.7s, international normalized ratio1.1; erythrocyte sedimentation rate 6 mm/h, liver function tests: aspartate aminotransferase 22.6 U/L, alanine transaminase 9.8 U/L, and ferritin level was 74.5 ng/ml.

At 00:20 on June 14th, 2020, the mother developed chills, felt feverish, cough, and complained of back pain. Her body temperature was 37.8°C. She was given oral paracetamol for fever and screened for infection. Although the possibility of intraamniotic infection (IAI) was raised, it was low‐grade fever (<38°C), and other findings supportive IAI like maternal leukocytosis, purulent cervical discharges, or fetal tachycardia were not present. She was continued on intravenous Ceftriaxone. No signs of respiratory distress were observed. Her oxygen saturation level was 98% on room air.

Nasopharyngeal swab for COVID‐19 was taken to exclude COVID‐19 infection at 04:30 the same day. Maternal GBS testing was negative. Placenta histopathology was not sent.

At 6:00, her temperature was 37.8°C, as well as mild dry cough and relative tachycardia. Respiratory rate was 20 breaths per minute chest radiography did not reveal any abnormality. Fortunately, at 8:30, her body temperature and tachycardia subsided, and fetal heart monitoring remained reassuring. Since the mother was suspected of having COVID‐19, universal surgical masking, and regular prevention measures for COVID‐19 were practiced by healthcare workers who attended the delivery room.

Following the local infection control guidelines, the delivery took place in the isolation room with no skin‐to‐skin contact between the mother and her infant.

The baby girl was delivered on June 14 at 17:00 via vaginal delivery without any complications. She was immediately separated from the mother and shifted to a single room where initial steps of resuscitation were applied. Apgar scores were 8, 9 and 9 at 1, 5, and 10 minutes, respectively. Physical examination revealed a well‐appearing baby.

Birth weight was 3070 grams (50th to 90th percentile), length of 52 cm (50th to 90th percentile), and her head circumference of 35 cm (50th percentile).

The newborn was admitted to the level‐II neonatal intensive care unit (NICU) in an isolation room with a continuous cardiac monitor.

Breastfeeding was prevented according to the local policy of infection control for suspected newborn cases for COVID‐19.

The infant's body temperature was 36.6°C, and her respiratory rate was 50 breaths per minute, with a heart rate of 144 beats per minute. Her oxygen saturation level was greater than 95% on room air.

On June 14th, at 20.00, a nasopharyngeal swab result taken from the mother at 04.30 June 14th reported positive for SARS‐CoV‐2 infection.

Nasopharyngeal swab sampling for the baby was taken at the age of 24 hours and also came positive. Her laboratory blood tests showed white blood cells 14.27 x109 cells /L, neutrophil 9.39 x109 cells/L, hemoglobin 17.4 g/dl, platelet 231x109/L, and hematocrit 57%; C‐reactive protein 3.11 (positive > 10 mg/L); blood gas analysis pH 7.4 with base excess–5.5; liver function tests: aspartate aminotransferase 16 U/L and alanine transaminase 2.9 U/L. Blood culture was negative. Chest radiography did not reveal any abnormality.

The baby continued to be asymptomatic and maintained normal vital signs in room air. She was tolerating regular formula feeding orally. A repeated nasopharyngeal swab done at 48 hours of life was negative. Both baby and mother were discharged home on day 5 with no complication. The baby was monitored until day 28 of life, and she remained asymptomatic.

## 3 DISCUSSION

Presently, the possibility of vertical transmission is a big question to neonatologists and obstetricians.

Based on most negative samples from vaginal discharge, nasopharyngeal swabs, amniotic fluid, or breast milk, the World Health Organization reports no vertical transmission evidence when COVID‐19 infection appears in pregnant women in the last trimester.^4^

This conclusion opposed the recently reported paper suggesting vertical transmission on preterm neonate delivered from the COVID‐19 positive mother under infection control and prevention measures where testing for reverse‐transcription polymerase chain reaction (RT‐PCR) of bronchoalveolar lavage, rectal swabs, amniotic fluid, blood, and nasopharyngeal swab in a neonate was positive for E and S genes COVID‐19 virus.^5^

Other reports also identified the presence of SARS‐CoV‐2 in the villous chorion and amniotic fluid.^6^

The index case is a term newborn baby with nasopharyngeal swab sample testing positive by RT‐PCR for SARS‐CoV‐2 infection at 24 hours after vaginal delivery.

Although the test's optimal timing is not precise, the Centers for Disease Control and Prevention suggest doing a test at 24 hours of age to avoid the possibility of false‐positive results due to contamination of the SARS‐CoV‐2 that may present in maternal fluids.^7^

Our case one of the few reported positive nasopharyngeal swab RT‐PCR performed at 24 hours of age. Though the possibility of cross‐infection should be considered in the presented case, it was doubtful due to the immediate separation of mother and daughter after birth and applied airborne, droplet, and contact transmission precautions. Delayed cord clamping was not done, and the neonate was kept in an incubator in a single room.

The possibility of the intrauterine vertical transmission of SAR ‐CoV‐2 infection was also demonstrated in other reports.^8^, ^9^, ^10^, ^11^, ^12^.

Zeng et al presented the report of neonates with positive pharyngeal tests two days after birth. Infants were symptomatic with the pneumonic findings in chest radiography that raised the possibility of vertical transmission of COVID‐19.^11^ Authors reported positive SARS‐CoV‐2 with nasopharyngeal and anal swab samples collected on the infants on day 2 of life with strict infection prevention and control during procedures done throughout the delivery. Almost all reported cases were delivered by the emergency cesarean section and had confirmed maternal SARS‐CoV‐2 infection.

Our case was born by vaginal labor, though the risk of cross‐infection during vaginal delivery is not clear, while Carosso et al suggest that vaginal delivery is one of the potential risk factors for vertical transmission because of contact with SARS‐CoV‐2 present in the maternal stool.^13^

Furthermore, Scorzolini et al observed a positive pharyngeal PCR test of vaginal fluid in a woman with COVID‐19 infection that provides the potential possibility of COVID‐19 vertical transmission in vaginal delivery^12^.

Interestingly, the authors observed initially negative PCR result of vaginal swab that turned into positive on day 7 and 20 after SARS‐CoV‐2 symptoms in the pregnant women that advocate cesarean section in case of maternal COVID‐19 infection.

A recently published report described neonate with positive nasopharyngeal, oropharyngeal swabs immediately after birth, where mother and baby stools were also positive for SARS‐CoV‐2, besides the viral RNA detected in the breast milk.^14^

Although, nasopharyngeal swab specimen for SARS‐CoV‐2 by RT‐PCR is considered one of the best ways to confirm COVID‐19; however, most studies did not reveal the presence of SARS‐CoV‐2 RNA in the specimens like amniotic fluid, cord blood, breast milk, or vaginal secretions. Udugama et al suggest that the results may be influenced by viral load, sample technique, or early disease stage.^15^ Those raise concerns about the real validity of a negative pharyngeal swab RT‐PCR test in infants born to COVID‐19 positive mothers.^16^ Therefore, negative RT PCR cannot rule out COVID‐19 infection.

Moreover, the evidence of vertical transmission of COVID‐19 was assessed by Ig M antibodies’ level in the infants from COVID‐19 mothers in several studies.

Dong et al reported elevated IgM antibodies to SARS‐CoV‐2 two hours after the birth in a neonate born to confirmed SARS‐CoV‐2 pregnant women.^17^ Also, elevated anti‐SARS‐CoV‐2 IgM antibodies were observed in two newborn infants in another recently published study suggesting the intrauterine infection,^18^ though, at the same time, several requested nasopharyngeal swabs for COVID‐19 were negative in both reports.

These serological results raise more concern about the risk of vertical transmission from mother to her baby. However, the rate of false‐positive results is significant in serologic studies and a challenging route to approach many congenital infections. Based on the latest update from the Centers for Disease Control and Prevention, at present serologic testing in detecting SARS‐CoV‐2 acute infection is limited and not recommended.^7^

During the whole hospital course, our patient was stable. Several studies in newborn infants with COVID −19, suggesting that infants usually have mild manifestations of the disease.^9^, ^10^, ^19^

However, several reported clinical analyses proposed that infants with SARS‐CoV‐2 infection may present with premature delivery, respiratory distress, gastrointestinal symptoms, and laboratory results abnormalities like elevated liver enzymes, thrombocytopenia, and increased mortality rate.^20^

We hypothesize that vaginal mode of delivery, PROM, and possible contact with a maternal stool that may contain SARS‐CoV‐2 may lead to vertical transmission of the virus in the presented case delivered in strict isolation mode with a positive pharyngeal swab at the age of 24 hours. However, our report is limited by the lack of mother's milk, amniotic fluid, and cord blood testing.

## 4 CONCLUSION

Our case is a probable case of congenital COVID‐19. However, for confirmation of congenital COVID‐19, more evidence is required to determine the route of vertical transmission. Variability of testing and findings raised the importance of universal approach protocol for suspected COVID‐19 neonates that would allow more optimal methods of diagnosis all over the world. Given the importance of vertical transmission, it is suggested to apply proper infection control and prevention measures, early separation, isolation, and screening neonates delivered from COVID‐19 positive mother with further close follow‐up.

## CONFLICT OF INTEREST

None to declare.

## AUTHOR CONTRIBUTIONS

RAH: collected the data, wrote the paper, and critically revised the article. LBM, HHS, and AAR: critically revised the article. OIH, MA, and AAR: collected the data and prepared the primary draft.

## ETHICAL STATEMENT

Informed consent was obtained from parents for reporting this case.

## Data Availability

The data that support the findings of this study are available on request from the corresponding author on the reasonable request.
